# Pulmonary Consumption

**Published:** 1842-10

**Authors:** 


ART. XIII.—*Pulmonary Consumption: its prevention and cure established on New Views of the Pathology of that Disease.* By HENRY GILBERT, M.R.C.S.—*London*, 1842. 8vo, pp. 296.

WITH the modesty commonly characteristic of the possessors of genius, Mr. Henry Gilbert commences his preface by "some apology" for adding another to the books already existing on the subject of pulmonary consumption. To us, who have read the volume, such apology is eminently needless; we hail with delight chapters as purely logical as they are profoundly meditative; especially as an under-current of sly humour (to which we are particularly partial) runs through them and leaves the critic "nothing to desire." The generous manner of noticing his inferiors in mental qualification and in fame is equally admirable and almost peculiar to Mr. Henry Gilbert; whether he kindly and patronisingly refers to "my highly talented friend, W. Farr, Esq.," gives occasional hint of the existence of such a person as "the ingenious Louis," or applies the rod of criticism to Sir James Clark, the *suaviter in modo*, and the placid condescension of a higher order of intelligence, alike conspicuously shine forth.

In the first chapter, that on the statistics of consumption, Mr. Gilbert refers to the difficulty which has hitherto existed in determining the influence of climate, quotes the conflicting opinions of writers on the point, and having thus strongly excited curiosity, gratifies it by the following luminous and decisive solution of that which previously had been little more than an unanswered riddle: "*My* opinion is, that till such time as the seeds of consumption are absorbed by the lacteals, climate can have no influence whatever in producing the disease, and it matters not whether it be hot or cold, moist or dry, changeable or regular." (p. 25.) This is rendered perfectly clear by Mr. Gilbert's remark in explanation: "The farmer is well aware that a warm temperature, with a moderate quantity of rain, encourages and favours the growth of corn, when once the seed is deposited in the earth; but he would never expect a favorable climate to raise grain till such time as the seed was sown." Of course.

It is interesting to know that "although phthisis is very prevalent in some warm countries," Mr. Gilbert "cannot assign heat as the cause, being clearly convinced, by the pathology of the disease, that it cannot be induced by this agent." It is likewise advisable the reader should be made aware of the author's "full conviction that a moist or damp atmosphere tends very materially to the relief of the phthisical, and that the disease is comparatively rare under such circumstances." Adopting Dr. Wells's notion, that "ague and consumption rarely occupy the same district," Mr. Gilbert gives the proposition all the energy—and truth—of poetry, asserting that "ague is the *daughter of humidity*, but over consumption she *rides rough-shod.*"

The influence of trades of various kinds on the development of consumption meets with investigation at Mr. Gilbert's hands. The most important point we find among the novelties of the section is the statement that Mr. Gilbert has not, "to the best of his recollection, seen a case of consumption among the trade of butchers for two years." Although we might have hesitated to credit Woolcombe, Young, &c. on this point, of the exemption of this class of subjects from the disease no doubt can henceforth be entertained.

The second chapter, that on the pathology of consumption, bears the old motto, "Felix qui potuit rerum cognoscere causas," which derives new life in its applicability to our author. Early in his inquiry Mr. Gilbert finds motive for lament in the infrequency with which we hear "of a confirmed case of pulmonary phthisis radically cured;" and in the fact that every case is commonly seen to fail "till nothing but a shadow remains for the cold earth to claim." He goes on to affirm, "it is reasonable to believe that there must be some cause for so universal a want of success," (p. 72;) in other words, where an effect exists there must be a cause. As it is to be presumed, however, that a change will come over the face of things, when Mr. Gilbert's pathology of the disease has become generally known, we hasten to impart this to the reader. The lacteals, according to him, absorb unorganizable matter, which circulates with the blood and is deposited in the lungs as tubercle. There can be no possible doubt of this, for it appears that "Dr. Ayre" tells us "plainly and distinctly that diseased mesenteric glands occur in children from the acrid condition of the duodenal contents;" and the author "*therefore sees* that unorganizable matter may be absorbed by the lacteals." The keenness of Mr. Gilbert's vision is as enviable as his reasoning is logical. Indeed "the force of reasoning could no further go:" the pathology of consumption is henceforth in the unvexed category of Q. E. D.

